# Dexamethasone Suppresses Palatal Cell Proliferation through miR-130a-3p

**DOI:** 10.3390/ijms222212453

**Published:** 2021-11-18

**Authors:** Hiroki Yoshioka, Goo Jun, Akiko Suzuki, Junichi Iwata

**Affiliations:** 1 Department of Diagnostic & Biomedical Sciences, School of Dentistry, The University of Texas Health Science Center at Houston, Houston, TX 77054, USA; Hiroki.Yoshioka@uth.tmc.edu (H.Y.); asg5p@umkc.edu (A.S.); 2 Center for Craniofacial Research, The University of Texas Health Science Center at Houston, Houston, TX 77054, USA; 3 Department of Epidemiology, The University of Texas Health Science Center at Houston, Houston, TX 77030, USA; Goo.Jun@uth.tmc.edu

**Keywords:** environmental factor, cleft palate, microRNA, gene regulation, cell proliferation, birth defect

## Abstract

Cleft lip with or without cleft palate (CL/P) is one of the most common congenital birth defects. This study aims to identify novel pathogenic microRNAs associated with cleft palate (CP). Through data analyses of miRNA-sequencing for developing palatal shelves of C57BL/6J mice, we found that miR-449a-3p, miR-449a-5p, miR-449b, miR-449c-3p, and miR-449c-5p were significantly upregulated, and that miR-19a-3p, miR-130a-3p, miR-301a-3p, and miR-486b-5p were significantly downregulated, at embryonic day E14.5 compared to E13.5. Among them, overexpression of the miR-449 family (miR-449a-3p, miR-449a-5p, miR-449b, miR-449c-3p, and miR-449c-5p) and miR-486b-5p resulted in reduced cell proliferation in primary mouse embryonic palatal mesenchymal (MEPM) cells and mouse cranial neural crest cell line O9-1. On the other hand, inhibitors of miR-130a-3p and miR-301a-3p significantly reduced cell proliferation in MEPM and O9-1 cells. Notably, we found that treatment with dexamethasone, a glucocorticoid known to induce CP in mice, suppressed miR-130a-3p expression in both MEPM and O9-1 cells. Moreover, a miR-130a-3p mimic could ameliorate the cell proliferation defect induced by dexamethasone through normalization of *Slc24a2* expression. Taken together, our results suggest that miR-130-3p plays a crucial role in dexamethasone-induced CP in mice.

## 1. Introduction

Cleft lip with/without cleft palate (CL/P) is a relatively common congenital birth defect in humans that affects approximately 1 in 700 newborns worldwide [1]. The palate is composed of the primary palate, which derives from posterior protrusion of nasal processes, and a pair of secondary palates, derived from the lateral protrusion of the maxillary processes. The development of the secondary palate in mammals includes palatal shelf growth, elevation of the palatal shelves, fusion between paired palatal shelves, disappearance of the medial epithelial seam, and intramembranous ossification of the palatal processes of the premaxilla and palatine bone [2]. Mice have been widely used in the study of palate development, since palate formation and the associated molecular mechanisms of mice are similar to that of humans and occur in a short period of time [3]. In mice, secondary palate development initiates at embryonic day 11.5 (E11.5) with the formation of tissue folds overlying the future palatal shelves within the oral cavity. Cranial neural-crest-derived mesenchymal cells proliferate within the maxillary processes to form the palatal primordium, which further enlarges to develop the palatal shelves. The palatal shelves continuously grow vertically along the sides of the tongue by E13.5 and then, approximately at E14.0, they elevate to a horizontal position above the tongue. At E14.5, the two palatal shelves meet and start to fuse each at the middle of the oral cavity. Finally, the medial epithelial seam disintegrates by either apoptosis, migration toward epithelial triangles at both oral and nasal sides, or epithelial–mesenchymal transition to complete palatal fusion by E16.5 (Supplementary Figure S1). Any failure of these processes leads to CP [3].

The etiology of CL/P is complicated, with both genetic and environmental factors involved as well as their interactions [4,5]. As for environmental factors, maternal exposure to smoking and alcohol consumption are known risk factors for CL/P [3]. In addition, several teratogens (e.g., phenytoin and toxins such as dioxins and heavy metals) are known to cause CP [4]. Environmental factors are thought to influence expression of non-coding RNAs including microRNAs (miRNAs), which are small RNAs with 21–25 nucleotides that regulate the expression of target genes at post-transcriptional level [6]. A number of miRNAs have been found in various species to play roles in a wide array of cellular functions during embryonic development, including palate development [7,8]. We have previously reported that overexpression of miR-374a, miR-133b, and miR-4680-3p inhibits cell proliferation in cultured human palatal mesenchymal cells [9]. In addition, exposure to *all-trans* retinoic acid (*at*RA) alters the expression of miR-106-5p [10] and miR-124-3p [11] in mouse embryonic palatal shelves. A growing amount of evidence shows that miRNAs play crucial roles in development and pathological conditions; therefore, it is important to identify how the expression of miRNAs is altered under specific conditions and in presence of chemicals known to cause of CP.

Dexamethasone (DEX) is a synthetic glucocorticoid (GC) clinically used for its anti-inflammatory and immunosuppressive actions through interference with various signaling pathways and molecules, including Toll-like receptors and mitogen-activated protein kinases [12]. GC signaling acts as either a transactivator or transrepressor of the target genes under physiological and pathological conditions. GCs in the extracellular fluid diffuse into the cytosol and bind to the GC receptor (GR) in the cytosol. In absence of GCs, nuclear protein GR forms a complex with heat shock protein 70 (HSP70), HSP90, FKBP52, and p23 in the cytosol. In presence of GCs, the GC–GR complex releases HSP70/HSP90/FKB92/p23 and forms a dimer of the GC–GR complex. The activated GC–GR dimer translocates into the nucleus and binds to the glucocorticoid response element (GRE) on the promoter region of its target genes, resulting in the activation of transcription (so called transactivation). In addition, the activated GC–GR complex binds to NFκB (p50/p65) without forming a dimer. This NFκB-conjoined monomeric complex represses transcription by binding to the NFκB response element instead of GRE. Thus, gene expression is suppressed (so called transrepression) [13,14]. Although GCs have tremendous therapeutic usefulness, they are also known for their teratogenicity and toxicity; for example, the oral or systemic administration of corticosteroids increases risk of CL/P two- to nine-fold, a risk of preterm birth or low birth weight [15,16,17], GC-induced osteonecrosis of the femoral head (GIONFH) [18], and GC-induced osteoporosis (GOI) [19]. Furthermore, DEX is known to penetrate the blood–placental barrier and bind to GR in the cytoplasm, causing CP in mice due to suppression of cell proliferation in the palatal mesenchyme [20,21], and craniofacial dysmorphism by upregulated *mmp13* expression in zebrafish [22,23]. Although GC treatment induces expression of both genes and miRNAs, the regulatory network of genes and miRNAs remains largely unknown.

Palate formation drastically changes between E13.5 and E14.5, from the cell proliferation phase to the differentiation and extracellular matrix (ECM) secretion phase. During this period, not only morphology but also the gene expression profile is altered according to cellular events. However, it is unclear how gene expression is regulated between E13.5 and E14.5 and whether altered miRNAs are associated with CP. In this study, we first searched for genes (and their functions) regulated by miRNAs during palate development using FaceBase datasets (https://www.facebase.org/, accessed on 27 May 2020). Through the analyses, we identified several miRNAs that were validated using mouse embryonic palatal mesenchymal (MEPM) cells and O9-1 cells, a mouse neural crest cell line. Furthermore, we evaluated whether DEX, *at*RA, and phenytoin, known teratogens which can induce CP in mice, influenced miRNA expression in MEPM and O9-1 cells.

## 2. Results

### 2.1. Genes and miRNAs Potentially Involved in Palate Development

Through secondary data analyses of the miRNA-seq and RNA-seq datasets available at FaceBase, we identified a total of nine miRNAs that were differentially expressed in the palate between E13.5 and E14.5, with a false discovery rate (FDR) < 0.05. A total of five miRNAs (miR-449a-3p, miR-449a-5p, miR-449b, miR-449c-3p, and miR-449c-5p) were upregulated, and a total of four miRNAs (miR-19a-3p, miR-130a-3p, miR-301a-3p, and miR-486b-5p) were downregulated at E14.5 compared to E13.5 (Supplementary Table S2). To identify genes anti-correlated with the expression of these miRNAs, we queried these miRNAs with four different sequence-based target prediction databases: TargetScan, mirDB, miRWalk, and miRTarBase (Supplementary Table S3).

### 2.2. miRNAs Involved in Cell Growth in MEPM and O9-1 Cells

First, we analyzed the expression of the identified miRNAs in MEPM and O9-1 cells, as well as in the palatal shelves, at E12.5 to E14.5. MiR-130a-3p was highly expressed in MEPM and O9-1 cells, while miR-301a-3p was expressed at moderate level; miR-449c-5p and miR-486b-5p were expressed at lower level, and miR-449c-3p was not detectable (Supplementary Table S4). miR-130a-3p was continuously downregulated through E12.5 to E14.5, miR-301a-3p and miR-486b-5p were transiently upregulated at E13.5, miR-449c-3p was detected at E14.5 only, and miR-449c-5p was upregulated at E14.5 compared to E12.5 and E13.5 (Supplementary Figure S2).

**Figure 1:**
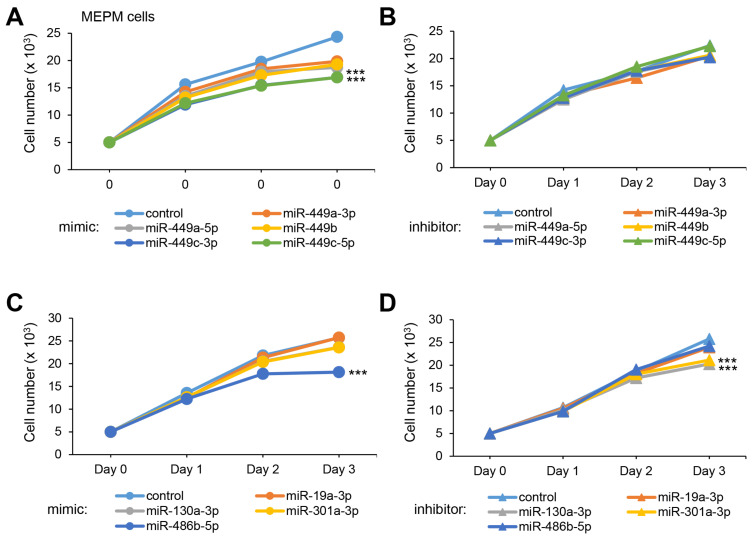
Effect of the predicted miRNAs on cell proliferation in MEPM cells. (**A**) Cell proliferation assays using MEPM cells from E13.5 palatal shelves treated with the indicated miRNA mimic: control, miR-449a-3p (*p* < 0.01), miR-449a-5p (*p* < 0.01), miR-449b (*p* < 0.01), miR-449c-3p (*p* < 0.001), and miR-449c-5p mimic (*p* < 0.001). *** *p* < 0.001. (**B**) Cell proliferation assays using MEPM cells treated with control and the indicated miR inhibitor: miR-449a-3p, miR-449a-5p, miR-449b, miR-449c-3p, and miR-449c-5p inhibitor. (**C**) Cell proliferation assays in MEPM cells treated with the control and the indicated miRNA mimic: miR-19a-3p, miR-130a-3p, miR-301a-3p, and miR-486b-5p (*p* < 0.001) mimic. *** *p* < 0.001. (**D**) Cell proliferation assays in MEPM cells treated with the indicated miRNA inhibitor, control, and miR-19a-3p, miR-130a-3p (*p* < 0.001), miR-301a-3p (*p* < 0.001), and miR-486b-5p inhibitor. *** *p* < 0.001. Each treatment group was compared to the negative control.

To test whether overexpression or downregulation of these miRNAs could influence cell growth (crucial at E13.5 for the growth of the palatal shelves), we conducted cell growth assays with a mimic and inhibitor for each miRNA and found that all five miR-449 family miRNAs significantly suppressed cell growth in both MEPM and O9-1 cells (Figure 1A and Figure S3A). Among them, miR-449c-3p and miR-449c-5p inhibited cell growth more than 30% in both MEPM and O9-1 cells. On the other hand, inhibitors for all of the miR-449 family did not affect cell growth in both MEPM and O9-1 cells (Figure 1B and Figure S3B). Moreover, we found that overexpression of miR-486b-5p and suppression of miR-130a-3p and miR-301a-3p inhibited cell growth (Figure 1C,D and Figure S3C,D).

**Figure 2:**
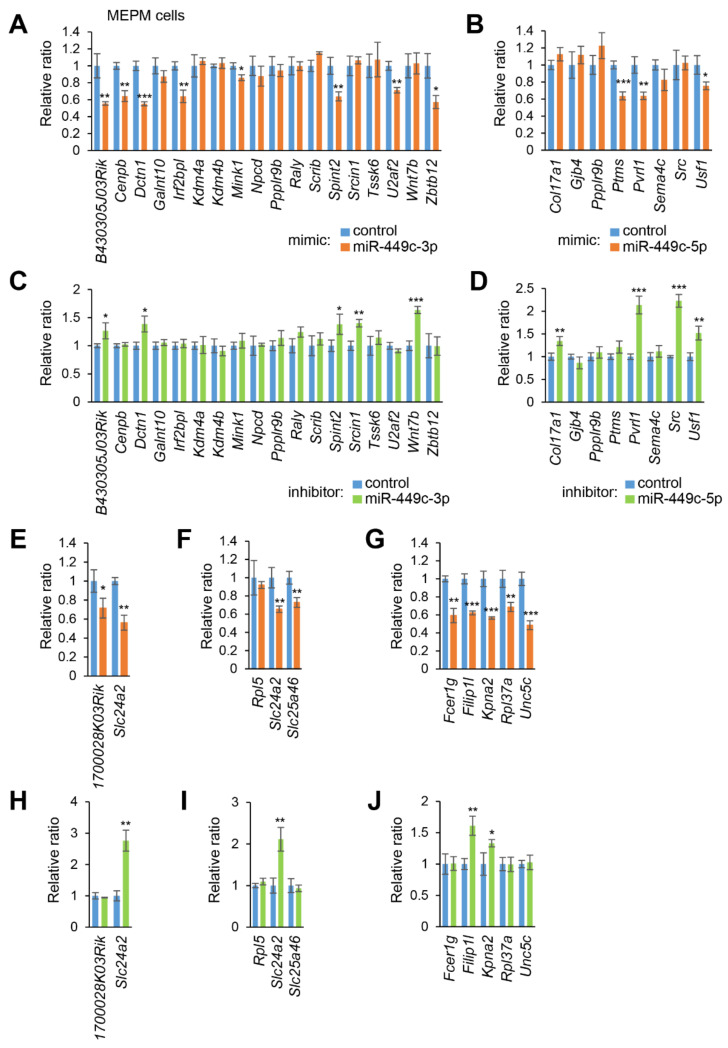
Effect of the predicted miRNAs on gene expression in MEPM cells. (**A**) Quantitative RT-PCR for treatment with miR-449c-3p mimic for 24 h in MEPM cells. (**B**) Quantitative RT-PCR for treatment with miR-449c-5p mimic for 24 h. (**C**) Quantitative RT-PCR for treatment with miR-449c-3p inhibitor for 24 h. (**D**) Quantitative RT-PCR for treatment with miR-449c-5p inhibitor for 24 h. (**E**) Quantitative RT-PCR for treatment with miR-130a-3p mimic for 24 h. (**F**) Quantitative RT-PCR for treatment with miR-301a-3p mimic for 24 h. (**G**) Quantitative RT-PCR for treatment with miR-486b-5p mimic for 24 h. (**H**) Quantitative RT-PCR for the miR-130a-3p inhibitor for 24 h. (**I**) Quantitative RT-PCR for treatment with miR-301a-3p inhibitor for 24 h. (**J**) Quantitative RT-PCR for the miR-486b-5p inhibitor treatment for 24 h. * *p* < 0.05; ** *p* < 0.01; *** *p* < 0.001. Each treatment group was compared to the negative control.

To identify genes regulated by either miR-449c-3p, miR-449c-5p, miR-130a-3p, miR-301a-3p, or miR-486b-5p, we conducted qRT-PCR analysis for the predicted genes and found that overexpression of miR-449c-3p significantly downregulated expression of *B43030503Rik*, *Cenpb*, *Dctn1*, *Irf2bpl*, *Mink1*, *Spint2*, *U2af2*, and *Zbtb12* in MEPM cells (Figure 2A), and of *B430305J03Rik*, *Cenpb*, *Dctn1*, *Irf2bpl*, *Mink1*, *Spint2*, *Srcin1*, *Tssk6*, *U2af2*, and *Zbtb12* in O9-1 cells (Supplementary Figure S4A). By contrast, suppression of miR-449c-3p significantly upregulated the expression of *B430305J03Rik*, *Dctn1*, *Spint2*, *Srcin1*, and *Wnt7b* in MEPM cells (Figure 2C), and of *B430305J03Rik*, *Cenpb*, *Dctn1*, *Kdm4a*, *Mink1*, *Npcd*, *Ppp1r9b*, *Scrib*, *Spint2*, *Srcin1*, *Tssk6*, *U2af2*, *Wnt7b*, and *Zbtb12* in O9-1 cells (Supplementary Figure S4C). Overexpression of miR-449c-5p significantly downregulated expression of *Ptms*, *Pvrl1*, and *Usf1* in MEPM cells (Figure 2B) and of *Ppplr9b*, *Ptms*, *Pvrl1*, *Sema4c*, and *Src* in O9-1 cells (Supplementary Figure S4B). Suppression of miR-449c-5p significantly upregulated the expression of *Col17a1*, *Pvrl1*, *Src*, and *Usf1* in MEPM cells (Figure 2D), and *Col17a1*, *Gjb4*, *Ppp1r9b*, *Nectin1*, *Sema4c*, and *Src* in O9-1 cells (Supplementary Figure S4D). Overexpression of miR-130a-3p significantly downregulated expression of *Slc24a2* and *1700028K03Rik* in both MEPM and O9-1 cells (Figure 2E and Figure S4E), and suppression of miR-130a-3p significantly upregulated *Slc24a2* expression in MEPM and O9-1 cells (Figure 2H and Figure S4H). Overexpression of miR-301a-3p downregulated *Slc24a2* and *Slc25a46* in MEPM cells (Figure 2F) and *Rpl5*, *Slc24a2*, and *Slc25a46* in O9-1 cells (Supplementary Figure S4F); suppression of miR-301a-3p upregulated *Slc24a2* expression in both MEPM and O9-1 cells (Figure 2I and Figure S4I). Overexpression of miR-486b-5p significantly downregulated expression of *Fcer1g*, *Filip1l*, *Kpna2*, *Rpl37a*, and *Unc5c* in MEPM cells (Figure 2G) and *Filip1l*, *Rpl37a*, and *Unc5c* in O9-1 cells (Supplementary Figure S4G), and suppression of miR-486b-5p significantly upregulated expression of *Filip1l* and *Kpna2* in MEPM cells (Figure 2J), and *Fcer1g*, *Filip1l*, *Kpna2*, *Rpl37a*, and *Unc5c* in O9-1 cells (Supplementary Figure S4J). Taken together, expression of *B430305J03Rik*, *Dctn1*, and *Spint2* for miR-449c-3p, *Pvrl1* for miR-449c-5p, *Slc24a2* for miR-130a-3p and miR-301a-3p, and *Filip1l* for miR-486b-5p was regulated in a dose-dependent manner. Currently, none of these candidate genes (Supplementary Table S3) have been reported as genes as related to CP [3]; information available from the CleftGeneDB database (https://bioinfo.uth.edu/CleftGeneDB, accessed on 27 May 2020). Therefore, we will analyze the function of these candidate genes in cell proliferation.

### 2.3. DEX Suppresses miR-130a-3p Expression in MEPM and O9-1 Cells

**Figure 3:**
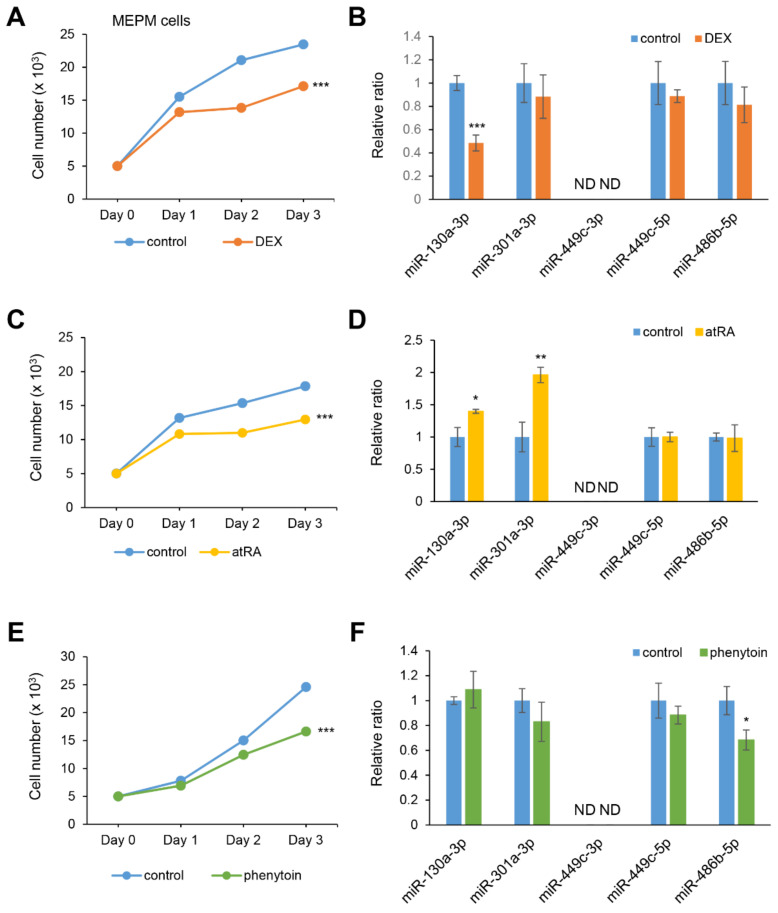
Influence of DEX, *at*RA, or phenytoin treatment on cell proliferation and gene expression in MEPM cells. (**A**,**C**,**E**) Cell proliferation assays in MEPM cells treated with 1 µM DEX (**A**), 10 µM *at*RA (**C**), or 50 μg/mL phenytoin (**E**) for 24, 48, and 72 h. *** *p* < 0.001 vs. control (*n* = 6). (**B**,**D**,**F**) Quantitative RT-PCR for the miR-130a-3p, miR-301a-3p, miR-449c-3p, miR-449c-5p, and miR-486b-5p after treatment with DEX (**B**), *at*RA (**D**), or phenytoin (**F**) for 72 h in MEPM cells. * *p* < 0.05; ** *p* < 0.01; *** *p* < 0.001. Each treatment group was compared to the control. ND; not detectable.

Excessive exposure to certain chemicals such as *at*RA, phenytoin, and DEX are known to cause CP in mice [4,24,25]. To investigate whether the expression of candidate miRNAs is associated with chemical exposure, we conducted cell growth assays in MEPM and O9-1 cells under treatment with either DEX, *at*RA, phenytoin, or vehicle. All three chemicals significantly suppressed cell growth in both MEPM and O9-1 cells (Figure 3A,C,E and Figure S5A,C,E). As we expected, miR-130a-3p expression was specifically inhibited under treatment with DEX (Figure 3B and Figure S5B). The expression of miR-130-3p and miR-301a-3p was upregulated under *at*RA treatment (Figure 3D and Figure S5D); the expression of miR-486b-5p was significantly suppressed under phenytoin treatment (Figure 3F and Figure S5F). The expression of miR-449c-5p was not altered and miR-449c-3p was not detected under the test conditions.

### 2.4. Overexpression of miR-130a-3p Restores Cell Proliferation under Treatment with DEX

**Figure 4:**
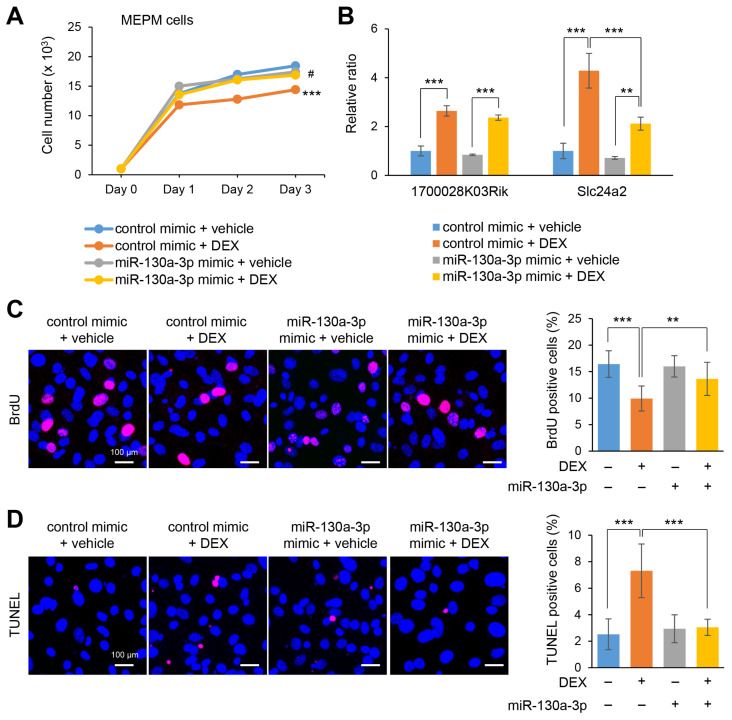
Effect of miR-130a-3p mimic against treatment with DEX on cell proliferation and gene expression in MEPM cells. (**A**) Cell proliferation assays in MEPM cells treated with DEX under miR-130a-3p mimic for 24, 48, or 72 h. *** *p* < 0.001 (control mimic + vehicle vs control mimic + DEX); ^#^ *p* < 0.05 (control mimic + DEX vs. miR-130a-3p mimic + DEX). (**B**) Quantitative RT-PCR for the *1700028K03Rik* and *Slc24a2* after treatment with DEX with miR-130a-3p mimic for 72 h in MEPM cells. ** *p* < 0.01; *** *p* < 0.001. (**C**) BrdU staining (red) in MEPM cells after 1 μM DEX treatment for 72 h. Nuclei were counterstained with DAPI (blue). Scale bar, 100 μm. Graph shows the quantification of BrdU-positive cells. ** *p* < 0.01; *** *p* < 0.001. (**D**) TUNEL staining (red) in MEPM cells after treatment with 1 μM DEX for 72 h. Nuclei were counterstained with DAPI (blue). Scale bar, 100 μm. Graph shows the quantification of TUNEL-positive cells. *** *p* < 0.001.

To evaluate the contribution of miR-130a-3p to cell growth under treatment with DEX, MEPM and O9-1 were treated with a miR-130a-3p mimic. Notably, the miR-130a-3p mimic completely normalized cell growth under treatment with dexamethasone (Figure 4A,C and Figure S6A,C). The upregulated expression of *Slc24a2*, but not *1700028K03Rik*, was partially restored by treatment with DEX, in MEPM and O9-1 cells (Figure 4B and Figure S6B). Thus, our results indicate that DEX can inhibit cell growth through downregulation of miR-130a-3p in MEPM and O9-1 cells. There are two possibilities for the suppression of cell growth: suppressed cell proliferation or increased cell death. We found that miR-130a-3p contributes to the suppression of cell growth through a decrease of cell proliferation and increase in apoptosis under DEX in both MEPM and O9-1 cells (Figure 4D and Figure S6D).

### 2.5. Slc24a2 Induces Apoptosis in MEPM and O9-1 Cells

**Figure 5:**
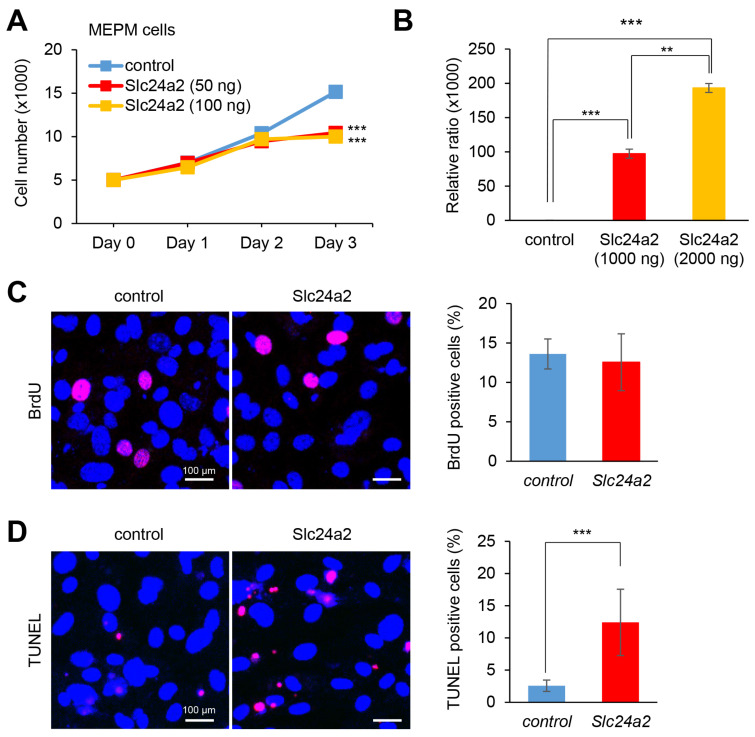
Overexpression of Slc24a2 inhibits cell proliferation activity in MEPM cells. (**A**) Cell proliferation assays in MEPM cells treated with 50 ng or 100 ng Slc24a2 for 24, 48, or 72 h. (**B**) Quantitative RT-PCR for the *Slc24a2* after treatment with *Slc24a2* DNA plasmid for 24 h in MEPM cells. ** *p* < 0.01; *** *p* < 0.001. (**C**) BrdU staining (red) in MEPM cells after transfecting 1 μg of *Slc24a2* DNA plasmid for 48 h. Nuclei were counterstained with DAPI (blue). Scale bar, 100 μm. Graph shows the quantification of BrdU-positive cells. (**D**) TUNEL staining (red) in MEPM cells after treatment with 1 μg *Slc24a2* DNA plasmid for 48 h. Nuclei were counterstained with DAPI (blue). Scale bar, 100 μm. Graph shows the quantification of TUNEL-positive cells. *** *p* < 0.001.

The role of *Slc24a2* in cell growth has not been evaluated before. Therefore, we tested the effect of overexpression of *Slc24a2* in MEPM and O9-1 cells and found that *Slc24a2* overexpression inhibited cell growth (Figure 5A and Figure S7A). To clarify the contribution of cell proliferation and apoptosis in cell growth inhibition, we performed BrdU incorporation (for cell proliferation) and TUNEL (for cell death) assays, under *Slc24a2* overexpression, in MEPM and O9-1 cells and found that *Slc24a2* overexpression induces apoptosis, but does not suppress cell proliferation (Figure 5C,D and Figure S7C,D). Taken together, our results indicate that DEX inhibits cell growth due to *Slc24a2*-mediated cell death through downregulation of miR-130a-3p in MEPM and O9-1 cells.

## 3. Discussion

miRNAs play a role in various diseases and development processes, including craniofacial development [3]. In this study, we attempted to identify miRNAs differentially expressed in the palatal shelves at E13.5 and E14.5, which are critical stages for palate development. We found that the miR-449 family was upregulated at E14.5 compared to E13.5, and miR-19a-3p, miR-130a-3p, miR-301a-3p, and miR-486b-5p were downregulated at E14.5 compared to E13.5. Among them, overexpression of the miR-449 family and miR-486b-5p mimic, and inhibition of miR-130a-3p and miR-301a-3p, suppressed cell proliferation in both MEPM and O9-1 cells. Therefore, miR-130a-3p, miR-301a-3p, the miR-449 family, and miR-486b-5p were considered as strong candidates for CP.

The miRNAs identified in this study have been reported in cancer research. Several studies suggest that miR-130a is important in the progression of several types of cancers and a potential oncogenic miRNA [26]. For example, miR-130a is upregulated in oral squamous cell carcinoma and suppresses expression of *TSC1*, a tumor suppressor gene, and an miR-301a inhibitor suppresses pancreatic tumor growth in a xenograft model [27]. Overexpression of miR-130a-3p promotes cell proliferation via negative regulation of Runt-related transcription factor 3 (RUNX3) in normal human cervical epithelial cells [28]. Inhibition of miR-130-3p represses cell proliferation by modulating the TGF-β type II receptor in gastric cancer cells [29]. In agreement with these results, miR-301a-3p inhibition suppressed cell proliferation in MEPM and O9-1 cells. miR-486-5p has been detected in various cancer cells [30,31], and its overexpression inhibits cell proliferation in leukemia cells, through targeting forkhead box protein O1 (FOXO1) [32], and accelerates anti-proliferative effects via PIM-1 in breast cancer cells [33]. The miR-449 family was first discovered in the embryonic mouse central nervous system [34]. The binding specificities of miR-449a and miR-449b are very similar, while miR-449c differs from those of others. All three miRNAs regulate the cell cycle and apoptosis. Overexpression of miR-449a induces cell cycle arrest in human bladder cancer cells [35] and suppresses cell proliferation through the regulation of cyclin D1 expression in colon cancers [36]. On the other hand, overexpression of miR-449c inhibits tumorigenesis in non-small cell lung cancer cells [37]. Since these miRNAs are associated with several signaling pathways, these miRNAs may play a crucial role in palate development through the regulation of these signaling pathways.

Currently, a total of 252 genes is reported as associated with CP in mice [3]. Detailed information is available at the CleftGeneDB database (https://bioinfo.uth.edu/CleftGeneDB, accessed on 27 May 2020). Among them, we found that *Trp63* is a CP-related gene for miR-449a-3p, *Ptprf* and *St14* for miR-449b, and *Scrib* for miR-449c-3p. Mice with a deficiency for the *Actn2*, *Alyref*, *Calm3*, *Dynll2*, *Galnt10*, or *Zfp740* genes regulated by miR-449a-3p, *Alyref* and *Zfp740* regulated by miR-449b, *B430305J03Rik*, *Galnt10*, and *Spint2* regulated by miR-449c-3p, *Filip1l* and *Rpl37a* regulated by miR-486b-5p, are not currently available. Among them, expression of *B430305J03Rik*, *Filip1l*, and *Spint2* was regulated by miR-449c-3p and miR-486b-5p in a dose-dependent manner in both MEPM and O9-1 cells. In this study, we found that overexpression of *Slc24a2* suppressed cell growth, and inhibition of miR-130a-3p and miR-301a-3p attenuated cell growth through upregulation of *Slc24a2*, a calcium transporter. Although mice with a deficiency for *Slc24a2* exhibit normal craniofacial development [38], overexpression of *Slc24a2* may therefore induce cell death via calcium overload.

Prenatal exposure to teratogens such as smoking, alcohol, and chemicals is also known to induce CP in laboratory animals and humans [4,5]. Excessive *at*RA, DEX, and phenytoin induce CP in mice [24,39,40]. Excessive *at*RA induces CP through upregulated miR-124-3p expression [11], and DEX induces miR-130b and miR-155 in porcine pre-adipocytes and differentiating 3T3-L1 pre-adipocytes, respectively [41,42]. DEX also inhibits miR-132 expression through TGF-β signaling in pancreatic cancer [43]. Since TGF-β signaling plays crucial roles in palate development [44], a cocktail of miR-132 and miR-130a-3p mimic might be more efficient than a mimic of each miRNA. In addition, the feedback loops between miRNAs and genes and the regulatory networks of miRNAs and genes (e.g., one miRNA regulates expression of multiple genes; gene expression is influenced by multiple miRNAs) may be involved in the rapid regulation of miRNAs by GCs.

In conclusion, we identified several miRNAs (miR-130a-3p, miR-301a-3p, miR-449a-3p, miR-449a-5p, miR-449b, miR-449c-3p, miR-449c-5p, miR-486b-5p) that were differentially expressed during mouse palate development. Among them, miR-130a-3p induced by DEX treatment leads to apoptosis through upregulation of *Slc24a2*. This study sheds light on the role of miRNA in CP induced by DEX.

## 4. Material and Methods

### 4.1. Bioinformatic Analysis

Datasets from miRNA-sequencing (miRNA-seq) and total RNA-sequencing (RNA-seq) obtained from the developing palate of E13.5 and E14.5 mouse embryos (E13.5 miRNA-seq (FB00000346 and FB00000665.01), E13.5 RNA-seq (FB00000278 and FB00000278.02), E14.5 miRNA-seq (FB00000494.01 and FB00000666.01), E14.5 RNA-seq (FB00000755.01, FB00000756.01, FB00000759.01, FB00000760.01, FB00000763.01, FB00000764.01, FB00000768.01, and FB00000769.91) available at FaceBase) were analyzed. All miRNA-seq data were analyzed after re-mapping all the miRNA fastq files using sRNAtoolbox [45] by first removing adapter sequences and barcodes with the “adapter = TCGTATGCCGTCTTCTGCTTG removeBarcode = 3” option, and then counting miRNAs by running “java -jar sRNAbench.jar microRNA = mmu” command. Replica samples from FB00000665.01 and FB00000666.01 with less than the minimum read were excluded from the analyses. All mRNA data were converted from the total RNA-seq FASTQ files using STAR aligner with “--runMode alignReads --outSAMtype BAM SortedByCoordinate --quantMode TranscriptomeSAM GeneCounts” options and with the GRCm38 reference sequence [46] and RSEM with “rsem-calculate-expression” option on the BAM file generated by STAR [47]. Differential expression analyses were conducted using the edgeR package [48], which includes the LIMMA (Linear Models for Microarray) package, by designating E13.5 and E13.5 as groups in the design matrix and using calcNormFactors, estimateGLMCommonDisp, estimateGLMTrendedDisp, estimateGLMTagwiseDisp, glmFit and glmLRT functions sequentially. *p*-values were adjusted for FDR using the Benjamini–Hochberg procedure, and FDR < 0.05 was used as threshold.

### 4.2. Cell Culture

MEPM cells were isolated from the palatal shelves of E13.5 C57BL/6J mice. Briefly, palatal shelves were dissected in D-PBS and suspended as single cells by 0.25% trypsin/0.05% EDTA (Sigma Aldrich, St. Louis, MO, USA) for 5 min at 37 °C. MEPM cells were maintained with Dulbecco’s modified Eagle’s medium (high glucose) (DMEM; Sigma Aldrich) supplemented with 10% fetal bovine serum (FBS), penicillin/streptomycin (Sigma Aldrich), β-mercaptoethanol (ThermoFisher Scientific, Waltham, MA, USA), and nonessential amino acids (Sigma Aldrich) at 37 °C in a humidified atmosphere with 5% CO_2_. O9-1 cells (SCC049, Sigma-Aldrich) were maintained in a conditioned medium provided from STO cells (a mouse embryonic fibroblast cell line; CRL-1503, ATCC), supplemented with 25 ng/mL basic fibroblast growth factor (R&D systems, Minneapolis, MN, USA), 1000 U/mL leukemia inhibitory factor (Sigma Aldrich), as previously described [49].

### 4.3. Cell Proliferation Assay

MEPM and O9-1 cells were plated onto 96-well plates at a density of 5000 cells (MEPM cells) or 1000 cells (O9-1 cells) per well and treated with a mimic for negative control (4464061, mirVana miRNA mimic, ThermoFisher Scientific, Waltham, MA, USA) or each miRNA (4464066, mirVana miRNA mimic, ThermoFisher Scientific), or an inhibitor for negative control (4464079, mirVana miRNA mimic, ThermoFisher Scientific) or each miRNA (4464084; mirVana miRNA inhibitor, ThermoFisher Scientific), as previously described [50]. For chemical treatment, cells were plated onto 96-well plates at a density of 5000 (MEPM cells) or 1000 cells (O9-1 cells) per well and treated with either 10 μM *at*RA (R2625, Sigma-Aldrich), 50 μg/mL phenytoin (D4505, Sigma-Aldrich), 1 μM DEX (D4902, Sigma-Aldrich), or vehicle after 6 h of seeding cells. After 24, 48, or 72 h of the treatment, cell numbers were counted, as previously described [50].

### 4.4. Quantitative RT-PCR

MEPM or O9-1 cells were plated at a density of 40,000 cells per dish. When the cells reached 80% confluence, they were treated with either mimic or inhibitor for each miRNA or negative control, as previously described [50]. After 24 h of transfection, total RNA was extracted with the QIAshredder and miRNeasy Mini Kit (QIAGEN, Hilden, Germany), according to the manufacturer’s protocol (*n* = 6 per group). For chemical treatments, both MEPM and O9-1 cells were treated with either 10 μM *at*RA, 50 μg/mL phenytoin, 1 μM DEX, or vehicle for 72 h (*n* = 3 per group). Extracted total RNAs were converted to cDNA and gene expression was analyzed, as previously described [50]. The PCR primers used in this study are listed in Supplementary Table S1. miRNA expression was measured, as previously described [50]. Probes for miR-130a-3p (mmu483331_mir), miR-449c-3p (mmu481842_mir), and miR-26a-5p (477995_mir) were purchased from Thermo Fisher Scientific. Probes for miR-301a-3p (MmiRQP0378), miR-449c-5p (MmiRQP1004), miR-486b-5p (MmiRQP0523), and U6 (MmiRQP9002) were purchased from GeneCopoeia.

### 4.5. BrdU Incorporation and TUNEL Assay

MEPM and O9-1 cells were plated at a density of 15,000/dish (MEPM cells) or 5000/dish (O9-1 cells) and treated with an overexpression vector for mock- [pcDNA3.1 (52535, Addgene, Watertown, MA, USA)] or full-length mouse *Slc24a2* (75199, Addgene) under treatment with DEX. After 48 h, the cells were incubated with 100 μg/mL BrdU (B5002, Sigma Aldrich) for 1 h; incorporated BrdU was detected with a rat monoclonal antibody against BrdU (ab6326; Abcam, Cambridge, UK, 1:1000). The Click-iT Plus TUNEL Assay with Alexa 594 (C10618, molecular probes, Thermo Fisher Scientific) was used to detect apoptotic cells, according to the manufacturer’s protocol. A total of 12 fields, which were randomly selected from three independent experiments, was used for the quantification of BrdU-positive and TUNEL-positive cells.

### 4.6. Statistical Analysis in Experiments

All experiments were performed independently three times. The statistical significance of the differences between two groups was evaluated using a two-tailed Student *t* test. Multiple comparisons were evaluated with one-way analysis of variance (ANOVA) adjusted by the post hoc Tukey–Kramer’s test. Cell proliferation was analyzed by two-way ANOVA adjusted by the Dunnett’s test (for control vs treated group) or Tukey–Kramer’s test (for multiple group comparison). A *p* value less than 0.05 was considered to be statistically significant. Data are represented as mean ± standard deviation in the graphs.

## Data Availability

Not applicable.

## Supplementary Materials

The following are available online at https://www.mdpi.com/article/10.3390/ijms222212453/s1.
