# Supplementary material for: Dexamethasone Suppresses Palatal Cell Proliferation through miR-130a-3p
Source: Int J Mol Sci. 2021 Nov 18;22(22):12453. doi: 10.3390/ijms222212453 (PMC8621257; doi:10.3390/ijms222212453)
Supplement: Supplementary file 1 [file ijms-22-12453-s001.zip › ijms-1450882-supplementary.pdf]

Supplementary Information

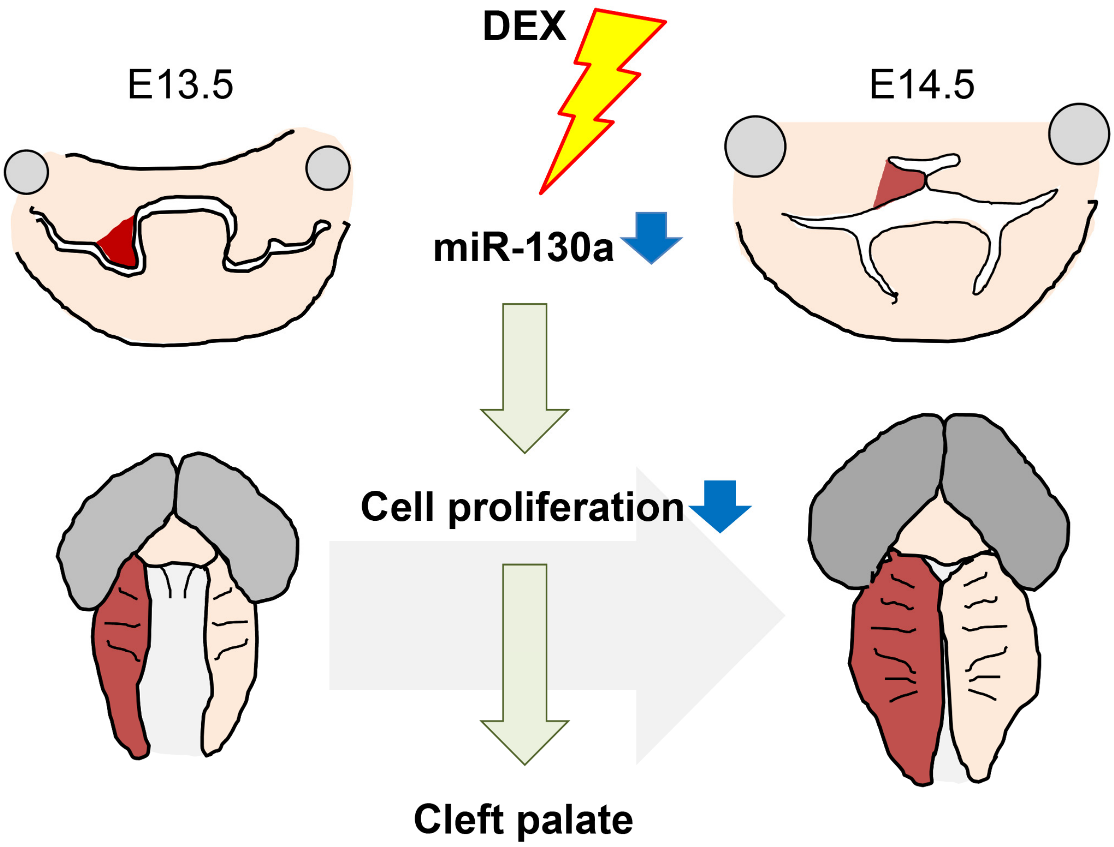

Figure S1. Schema of the effect of dexamethasone on mouse palate development. The palatal shelves, which give rise to the secondary palate (brown), first grow vertically along the lateral sides of the tongue. During E13.5–E14.5, the palatal shelves elevate into a horizontal position above the dorsum of the tongue, following the growth of the jaws and descent of the tongue. The elevated palatal shelves continuously grow toward each other and meet at the facial midline. Dexamethasone (DEX) suppresses miR-130a expression, which can suppress cell proliferation and lead to cleft palate.

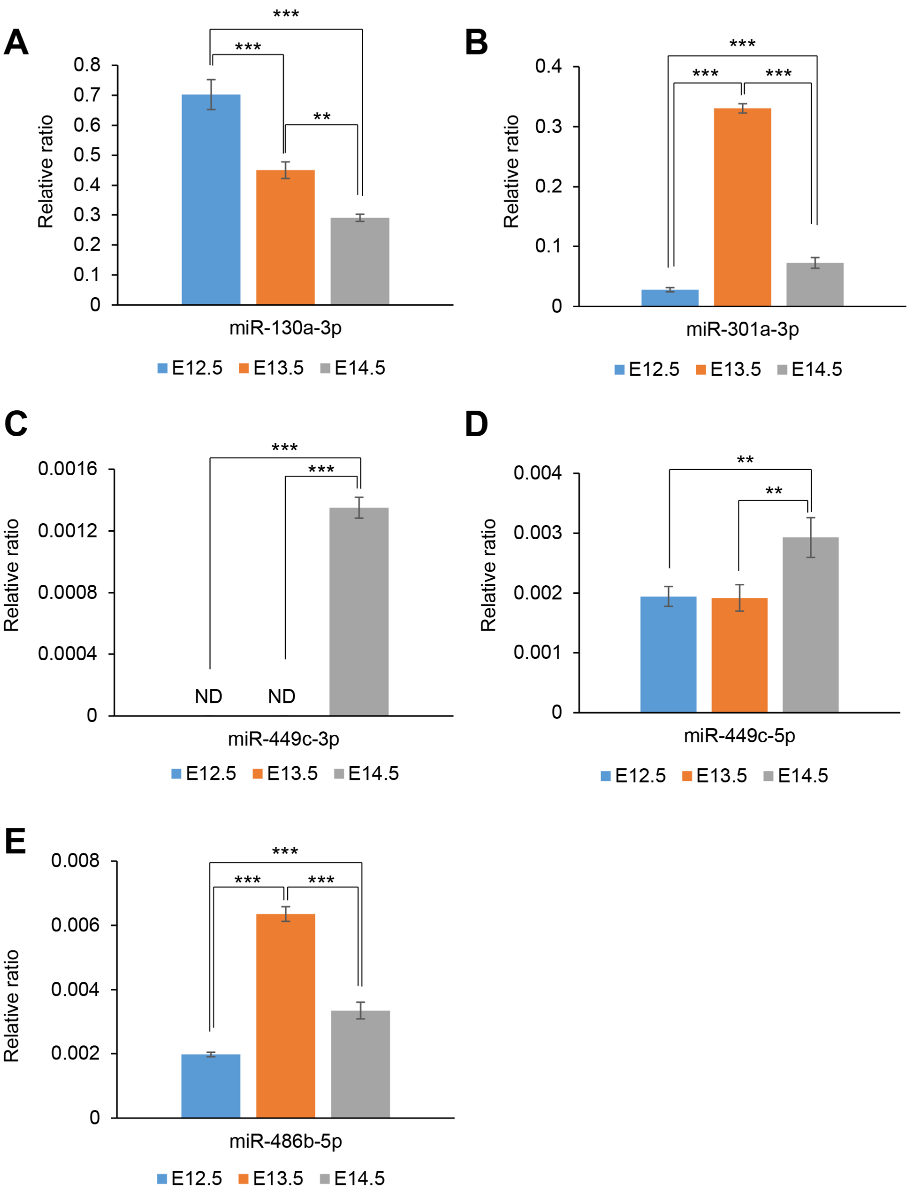

Figure S2. Physiological expression level of five miRNAs in the palatal shelf at E12.5, E13.5, and E14.5. (**A–E**) Quantitative RT-PCR for *miR-130a-3p* (A), *miR-301a-3p* (B), *miR-449c-3p* (C), *miR-449c-5p* (D), and *miR-486b-5p* (E) at E12.5, E13.5, and E14.5 in the palatal shelf. $**p < 0.01$; $***p < 0.001$. ND, not detectable.

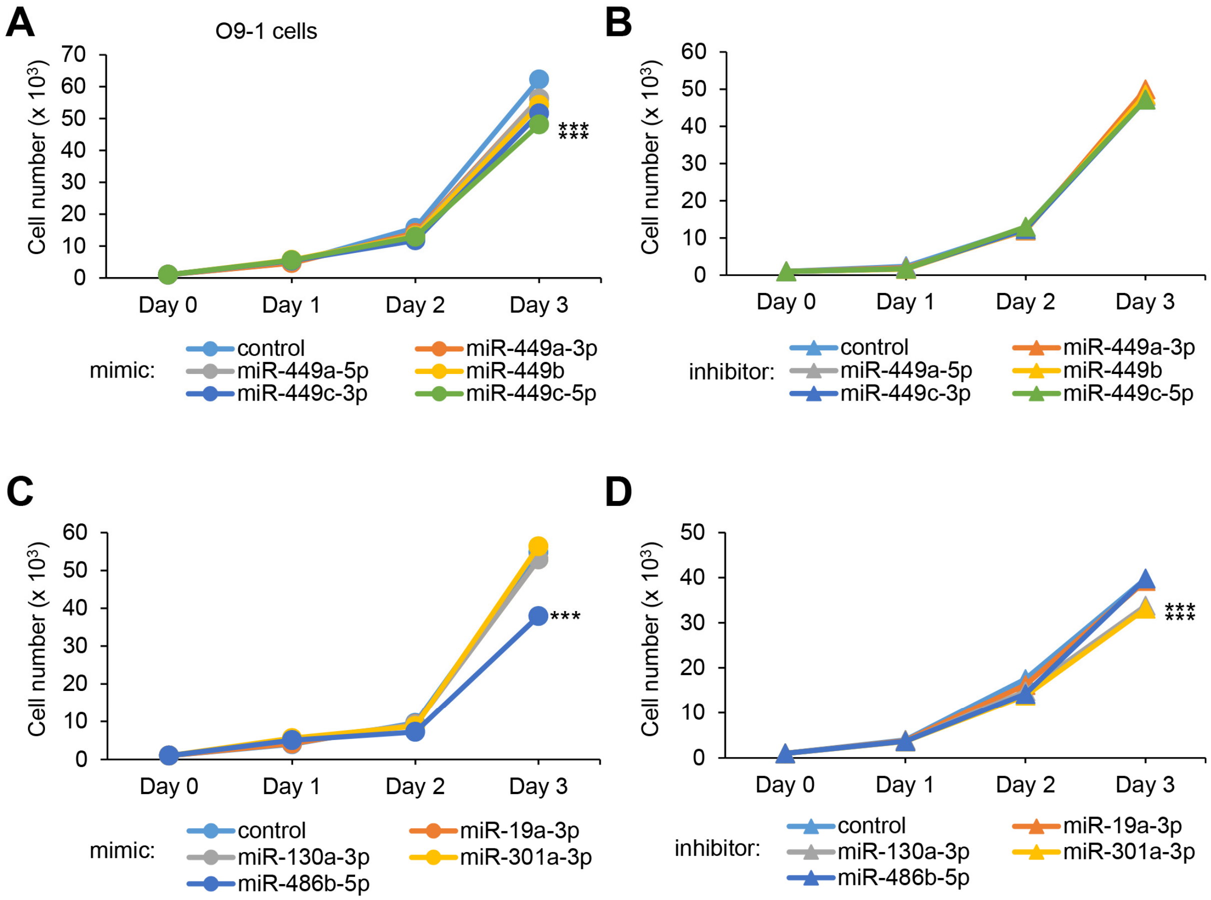

Figure S3. Effect of the predicted miRNAs on cell proliferation in O9-1 cells. (**A**) Cell proliferation assays using O9-1 cells from E13.5 palatal shelves treated with control or the indicated miR mimic: miR-449a-3p (p < 0.05), miR-449a-5p (p < 0.05), miR-449b (p < 0.01), miR-449c-3p (p < 0.001), and miR-449c-5p (p < 0.001) mimic. ***p < 0.001. (**B**) Cell proliferation assays using O9-1 cells treated with control or the indicated miR inhibitor: miR-449a-3p, miR-449a-5p, miR-449b, miR-449c-3p, and miR-449c-5p inhibitor. (**C**) Cell proliferation assays using O9-1 cells treated with control or the indicated miRNA mimic: miR-19a-3p, miR-130a-3p, miR-301a-3p, and miR-486b-5p mimic. (**D**) Cell proliferation assays using O9-1 cells treated with control or the indicated miRNA inhibitor: miR-19a-3p, miR-130a-3p, miR-301a-3p, and miR-486b-5p inhibitor. *p < 0.05; **p < 0.01; ***p < 0.001. Each treatment group was compared to the negative control.

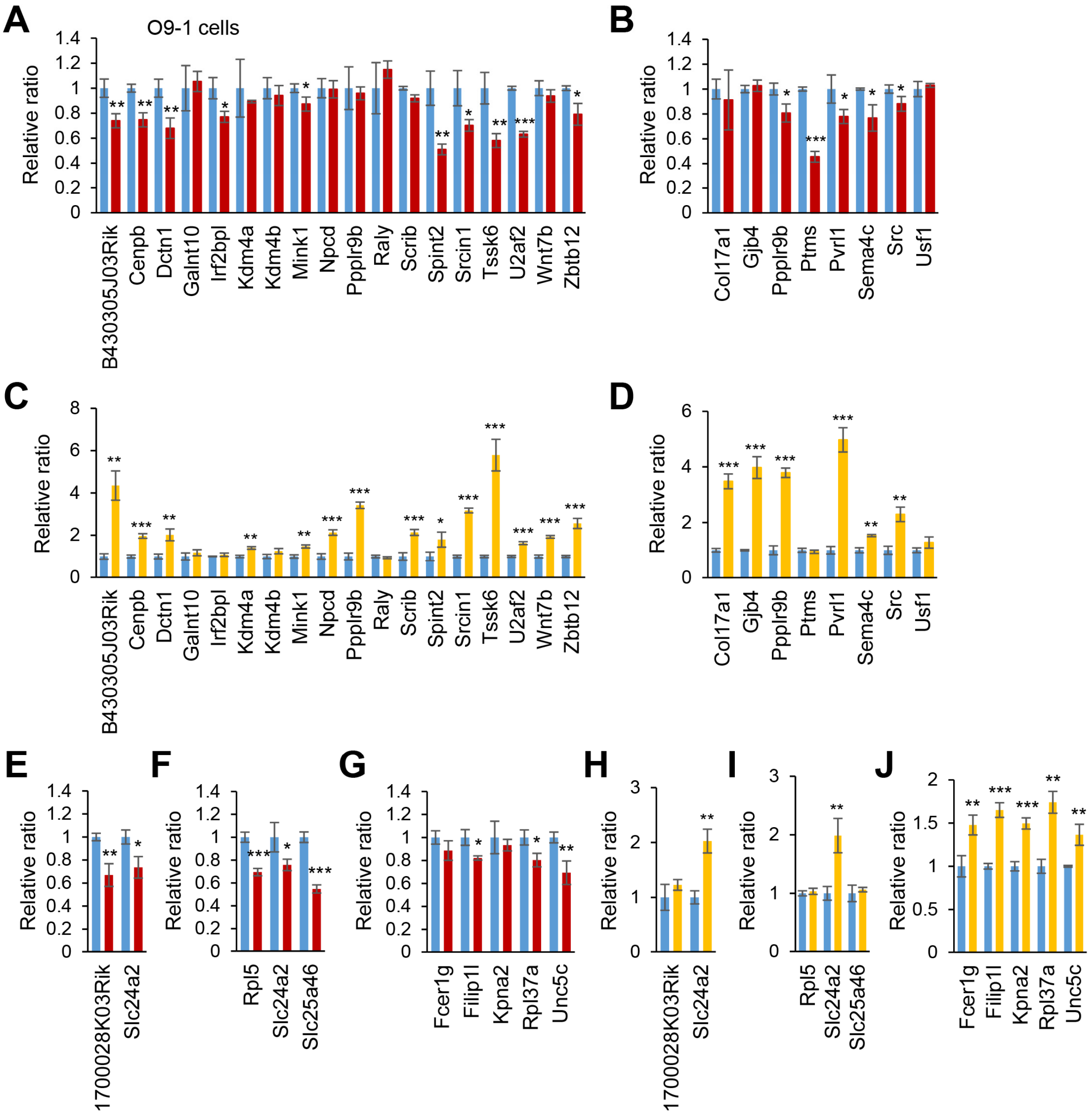

Figure S4. Effect of the predicted miRNAs on gene expression in O9-1 cells. (**A**) Quantitative RT-PCR for miR-449c-3p mimic treatment for 24 hours in O9-1 cells. (**B**) Quantitative RT-PCR for miR-449c-5p mimic treatment for 24 hours. (**C**) Quantitative RT-PCR for treatment with miR-449c-3p inhibitor for 24 hours. (**D**) Quantitative RT-PCR for treatment with miR-449c-5p inhibitor for 24 hours. (**E**) Quantitative RT-PCR for treatment with miR-130a-3p mimic for 24 hours. (**F**) Quantitative RT-PCR for treatment with miR-301a-3p mimic for 24 hours. (**G**) Quantitative RT-PCR for treatment with miR-486b-5p mimic for 24 hours. (**H**) Quantitative RT-PCR for treatment with miR-130a-3p inhibitor for 24 hours. (**I**) Quantitative RT-PCR for treatment with miR-301a-3p inhibitor for 24 hours. (**J**) Quantitative RT-PCR for treatment of miR-486b-5p inhibitor for 24 hours. $*p < 0.05$, $**p < 0.01$, $***p < 0.001$. Each treatment group was compared to the negative control.

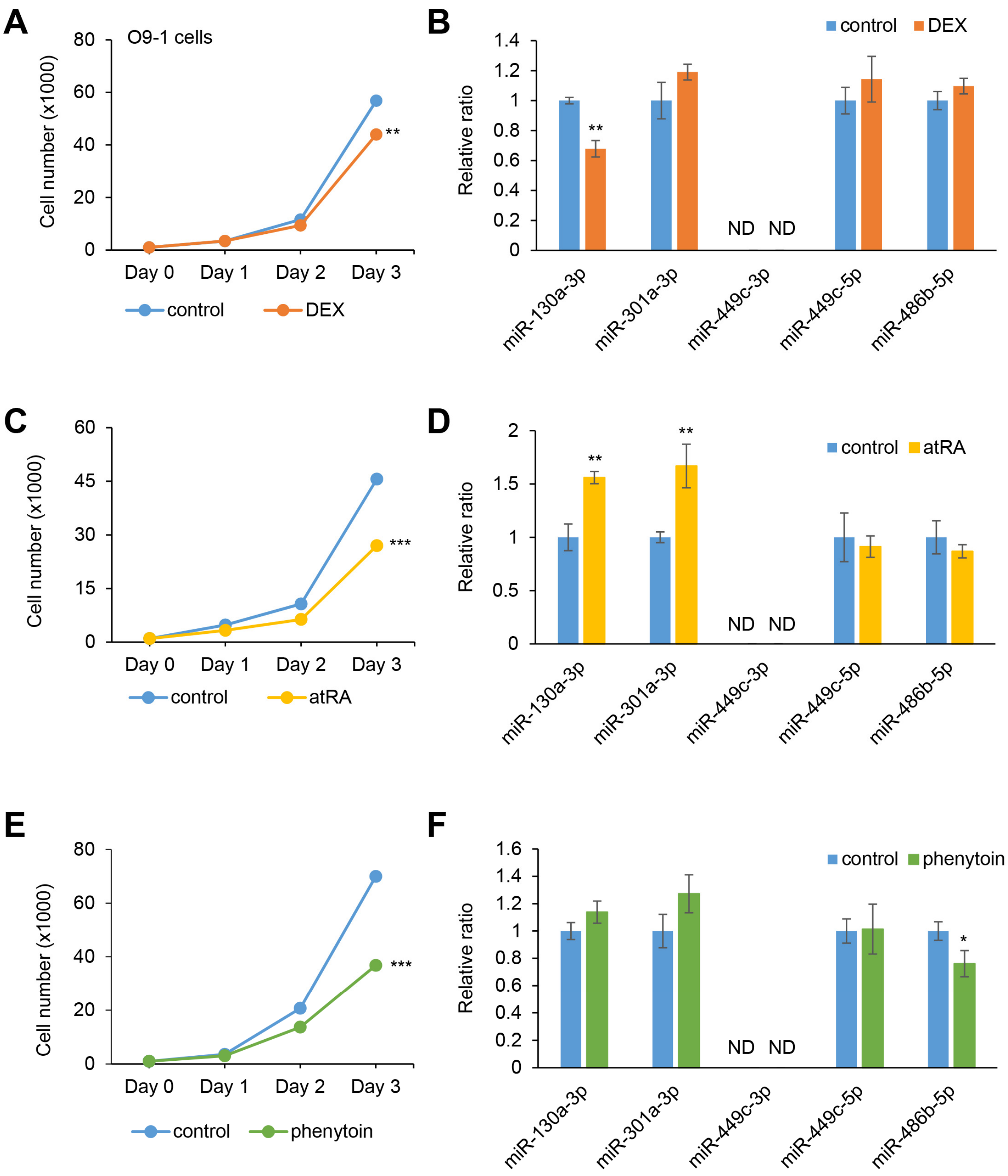

Figure S5. Influence of dexamethasone (DEX), *at*RA, or phenytoin treatment on cell proliferation and gene expression in O9-1 cells. (**A–D**) Cell proliferation assays in O9-1 cells treated with 1 µM DEX (A), 10 µM *at*RA (C), or 50 µg/mL phenytoin (E) for 24, 48, and 72 hours. $^{**}p < 0.01$; $^{***}p < 0.001$ vs control (n=6). Quantitative RT-PCR for miR-130a-3p, miR-301a-3p, miR-449c-3p, miR-449c-5p, and miR-486b-5p after treatment with DEX (B), *at*RA (D), or phenytoin (F) for 72 hours and in O9-1 cells. $^{*}p < 0.05$; $^{**}p < 0.01$. Each treatment group was compared to the control. ND; not detectable.

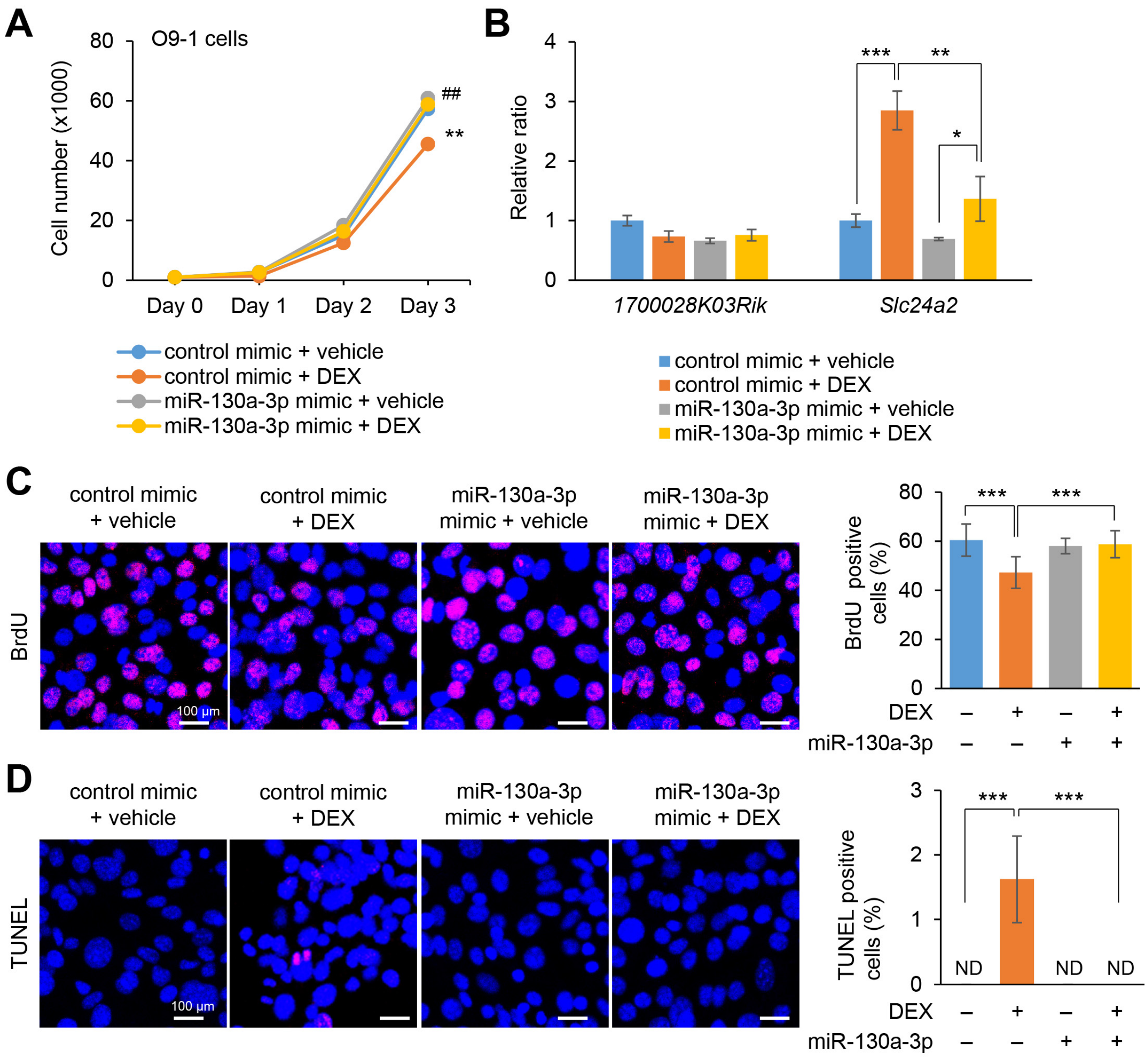

Figure S6. Effect of miR-130a-3p mimic against dexamethasone (DEX) treatment on cell proliferation and gene expression in O9-1 cells. (**A**) Cell proliferation assays in O9-1 cells treated with DEX under miR-130a-3p mimic for 24, 48, or 72 hours. $^{**}p < 0.01$ (control mimic + vehicle vs control mimic + DEX), $^{\#\#}p < 0.01$ (control mimic + DEX vs miR-130a-3p mimic + DEX). (**B**) Quantitative RT-PCR for *1700028K03Rik* and *Slc24a2* after treatment with DEX with miR-130a-3p mimic for 72 hours in O9-1 cells. $^{*}p < 0.05$; $^{**}p < 0.01$. (**C**) BrdU staining (red) in O9-1 cells after treatment with 1 µM DEX for 72 hours. Nuclei were counterstained with DAPI (blue). Scale bar, 100 µm. Graph shows the quantification of BrdU-positive cells. $^{***}p < 0.001$. (**D**) TUNEL staining (red) in O9-1 cells after treatment with 1 µM DEX for 72 hours. Nuclei were counterstained with DAPI (blue). Scale bar, 100 µm. Graph shows the quantification of TUNEL-positive cells. $^{***}p < 0.001$. ND; not detectable.

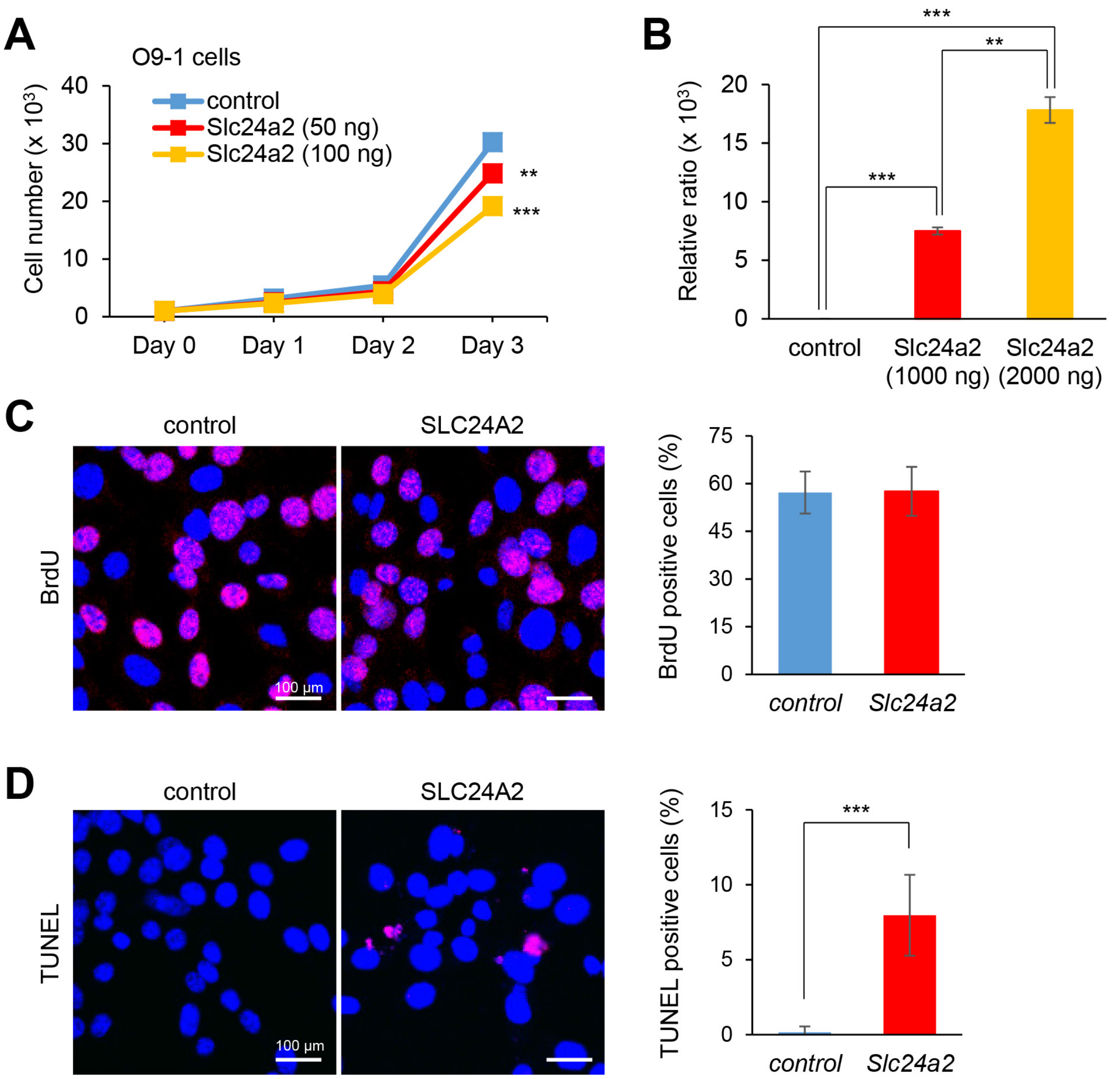

Figure S7. Overexpression of Slc24a2 inhibits cell proliferation activity in O9-1 cells. (**A**) Cell proliferation assays in O9-1 cells treated with 50 ng or 100 ng of Slc24a2 for 24, 48, or 72 hours. $^{**}p < 0.01$ vs control; $^{***}p < 0.001$ vs control. (**B**) Quantitative RT-PCR for *Slc24a2* after treatment with *Slc24a2* DNA plasmid for 24 hours in O9-1 cells. $^{**}p < 0.01$; $^{***}p < 0.001$. (**C**) BrdU staining (red) in O9-1 cells after transfecting 1 μg of *Slc24a2* DNA plasmid for 48 hours. Nuclei were counterstained with DAPI (blue). Scale bar, 100 μm. Graph shows the quantification of BrdU-positive cells. (**D**) TUNEL staining (red) in O9-1 cells after transfection of 1 μg *Slc24a2* DNA plasmid for 48 hours. Nuclei were counterstained with DAPI (blue). Scale bar, 100 μm. Graph shows the quantification of TUNEL-positive cells. $^{***}p < 0.001$.

Table S1. Primer sets used in this study.

Gene	Forward Primer	Reverse Primer
1700028K03Rik	5'-GGCAATGGATGAGCGTAGAGG-3'	5'-TATCAGAATACCGAACCTTGTGGC-3'
B430305J03Rik	5'-GGTTCGTGCTCCCACTTGG-3'	5'-CTTGCAAGCCCCAAACCTGC-3'
Cenpb	5'-AAGTACGGAGTGGCCTCCAC-3'	5'-GAAGTCGTCCATGCCCAGC -3'
Col17a1	5'-CTCCCCTGAGTACCCACGAAAG-3'	5'-CTACGGTGCCTTTCTTGGGGAT-3'
Dctn1	5'-ATGAGTACGGAGGCAAGCG-3'	5'-AGAATCACGCCCACCCATTTG-3'
Fcer1g	5'-ATCTCAGCCGTGATCTTGTTCT-3'	5'-ACCATACAAAAACAGGACAGCAT-3'
Filip1l	5'-AGCACTCAGTCGGCAAATTGA-3'	5'-AGCCTCTTATTGAGGTCTCTGC-3'
Galnt10	5'-AAGGAGGCTATCAGGAGGGAC-3'	5'-AGAGAGCGATTCAGGGAGATT-3'
Gjb4	5'-CAGACAAGCCGGCACCG-3'	5'-TCCCTGGAGAAATCCCCAGTTC-3'
Irf2bpl	5'-GTGTCCTCGTCCCGAAGAC-3'	5'-AATTGCCGCAACCACGGCATA-3'
Kdm4a	5'-GACATAGTGAGTCAGGACTGTCT-3'	5'-GGCCACAAACTTAGCCCCATA-3'
Kdm4b	5'-GGCGCAGTGGAACATCGG-3'	5'-CCGTGCTCCGGTGGTATG-3'
Kpna2	5'-ATG TCCACGAACGAGAATGCT-3'	5'AAGGAGCTGACGTTTCTTCTTTT -3'
Krt15	5'-TGATTTTGGTGGTGGCGATGG-3'	5'-AGTTGTCAATGGTGGCAGCC-3'
Mink1	5'-CCACCTACTATGGGGCCTTTA-3'	5'-AGCACCGCAGAACTCCATC-3'
Npcd	5'-GCCCTGCACTCCAAGATGG-3'	5'-GCCACATGCAGGCAGTGAAG-3'
Ppp1r9b	5'-CTCGAAGCTGGTCACTTCCG-3'	5'-TCCCCACTTTCTTCAACCTCC-3'
Pvrl1	5'-GACTCCATGTATGGCTTCATCG-3'	5'-CACTCGTTTCTCGTAGGGAGG -3'
Raly	5'-ATTCAGACCAGCAATGTAACCAA-3'	5'-CACAGAGCAACCAGCCACT-3'
Rpl37a	5'-GCTAAACGCACCAAGAAGGTC-3'	5'-CCACCGGCCACTGTTTTCAT-3'
Rpl5	5'-GGCGGCGAGAGGGTAAAAC-3'	5'-GCACAGACGATCATATCCCCTTC-3'
Scrib	5'-GTGGAGTCGGTGGATAAGCG-3'	5'-TGTAGCGGTAAATCTCCTCTGG-3'
Sema4c	5'-GAAGAGCAACCAGACCGAATG-3'	5'-ATAGGTGCCGCAGACATACAG-3'
Slc24a2	5'-GACAAGATTCGAGATTACACCCC-3'	5'-TGCAGAATGATGGCACCTTTC-3'
Slc25a46	5'-CCGAGCAGCGAACAGTTGAA-3'	5'-CGGCGTAAAACAATGCAAGGA-3'
Spint2	5'-TCCTCGCCTTGGTAGCTTCG-3'	5'-CAAATGGCTGGCAGGACCC-3'
Srcin1	5'-CAGAACCAGGAGTCGGTGC-3'	5'-GCCTTCTCTTCCAACTCAGGAC-3'
Tssk6	5'-GTGAAAGTGGCCACCTCCAAG-3'	5'-GCTGCCTCCATCACGATGTAC-3'
U2af2	5'-GGGATAGGAGACGACGAAGC-3'	5'-GGGGAGAACGAATCAATCCAC-3'
Unc5c	5'-CAGATGCTTGTGTTACCTGCC-3'	5'-GGTTTCTGGGAGTTCGTGAAAA-3'
Usf1	5'-TACCCTGCCACTCAGTCTATG-3'	5'-GCGGTGCTGGGGAAATATGTA-3'
Wnt7b	5'-CAGATGGGCATCGACGAGTG-3'	5'-CAGATTGCCCTGGCTGCAG-3'
Zbtb12	5'-TCCTCCGGGACCAGTTCTT-3'	5'-TCCACTACATGCTCCATTTGC-3'
Gapdh	5'-AACTTTGGCATTGTGGAAGG-3'	5'-ACACATTGGGGGTAGGAACA-3'

Table S2. miRNAs differentially expressed between E13.5 and E14.5.

miRNA	logFC	*P* value	FDR
**mmu-miR-449a-3p**	9.49	4.80 x 10^{10}	3.12 x 10^{7}
**mmu-miR-449a-5p**	6.65	4.63 x 10^{9}	2.00 x 10^{6}
**mmu-miR-449b**	8.18	3.69 x 10^{6}	7.98 x 10^{4}
**mmu-miR-449c-3p**	8.07	2.50 x 10^{7}	6.50 x 10^{5}
**mmu-miR-449c-5p**	6.14	4.42 x 10^{8}	1.43 x 10^{5}
**mmu-miR-19a-3p**	-3.53	2.49 x 10^{4}	4.04 x 10^{2}
**mmu-miR-130a-3p**	-3.34	3.00 x 10^{4}	4.32 x 10^{2}
**mmu-miR-301a-3p**	-3.61	1.84 x 10^{4}	3.41 x 10^{2}
**mmu-miR-486b-5p**	-15.33	4.03 x 10^{17}	5.24 x 10^{14}

Table S3. List of mRNAs that are predicted targets of miRNAs.

miRNA	Predicted target mRNAs
**mmu-miR-449a-3p**	*Actn2*, *Aldh2*, *Alyref*, *Calm3*, *Cbx6*, *Ccdc8*, *Dctn1*, *Ddx23*, *Dynll2*, *Ehmt2*, *Galnt10*, *Gcom1*, *Gjb6*, *Gpc1*, *Hap1*, *Igf2bp1*, *Lgi2*, *Lzts2*, *Map3k9*, *Mdfi*, *Mink1*, *Nyap1*, *Plekha7*, *Ppp1r9b*, *Raly*, *Slc8a2*, *Tnk1*, *Trp63*, *Zc3h18*, *Zfp740*
**mmu-miR-449a-5p**	*Itgb4*, *Ptms*, *Nectin1*, *Sema4c*, *Sox4*, *Src*, *Usf1*
**mmu-miR-449b**	*Alyref*, *Ddx23*, *Gigyf1*, *Map3k9*, *Plch2*, *Ptms*, *Ptprf*, *Nectin1*, *Sema4c*, *Src*, *St14*
**mmu-miR-449c-3p**	*B430305J03Rik*, *Cenpb*, *Dctn1*, *Galnt10, Irf2bpl*, *Kdm4a*, *Kdm4b*, *Mink1*, *Npcd*, *Ppp1r9*b, *Raly*, *Scrib*, *Spint2*, *Srcin1*, *Tssk6*, *U2af2*, *Wnt7b*, *Zbtb12*
**mmu-miR-449c-5p**	*Col17a1*, *Gjb4*, *Krt15*, *Ppp1r9b*, *Ptms*, *Nectin1*, *Sema4c*, *Src*, *Usf1*
**mmu-miR-19a-3p**	*Evi2b*, *Zfp871*
**mmu-miR-130a-3p**	*1700028K03Rik*, *Slc24a2*
**mmu-miR-301a-3p**	*Rpl5*, *Slc24a2*, *Slc25a46*
**mmu-miR-486b-5p**	*Fcer1g*, *Filip1l*, *Kpna2*, *Rpl37a*, *Unc5c*

Table S4. miRNA expression levels in MEPM and O9-1 cells.

	**MEPM cells**	**O9-1 cells**
miR-130a-3p/miR-26a-5p	$3.96 \pm 0.24 \times 10^{-1}$	1.04 ± 0.23
miR-301a-3p/U6	$1.62 \pm 0.27 \times 10^{-2}$	$3.52 \pm 0.43 \times 10^{-2}$
miR-449c-3p/miR-26a-5p	N.D.	N.D.
miR-449c-5p/U6	$3.84 \pm 0.71 \times 10^{-3}$	$5.37 \pm 0.47 \times 10^{-3}$
miR-486b-5p/U6	$7.14 \pm 0.81 \times 10^{-3}$	$1.59 \pm 0.11 \times 10^{-4}$
